# Supplementary material for: Defect-gradient-induced Rashba effect in van der Waals PtSe2 layers
Source: Nat Commun. 2022 May 19;13:2759. doi: 10.1038/s41467-022-30414-4 (PMC9120180; doi:10.1038/s41467-022-30414-4)
Supplement: Supplementary file 1 — Supplementary Information [file 41467_2022_30414_MOESM1_ESM.pdf]

Supplementary Information

Defect-gradient-induced Rashba effect in van der Waals $PtSe_2$ layers

Junhyeon Jo[1,6], Jung Hwa Kim[2,6], Choong H. Kim[3,4,6], Jaebyeong Lee[1,6], Daeseong Choe[1], Inseon Oh[1], Seunghyun Lee[1], Zonghoon Lee[1,2*], Hosub Jin[5*], & Jung-Woo Yoo[1*]

[1]Department of Materials Science and Engineering, Ulsan National Institute of Science and Technology, Ulsan 44919, Republic of Korea

[2]Center for Multidimensional Carbon Materials, Institute for Basic Science (IBS), Ulsan 44919, Republic of Korea

[3]Center for Correlated Electron Systems, Institute for Basic Science (IBS), Seoul 08826, Republic of Korea

[4]Department of Physics and Astronomy, Seoul National University, Seoul 08826, Republic of Korea

[5]Department of Physics, Ulsan National Institute of Science and Technology, Ulsan 44919, Republic of Korea

[6]These authors contributed equally: Junhyeon Jo, Jung Hwa Kim, Choong H. Kim, Jaebyeong Lee.

*Corresponding author email: zhlee@unist.ac.kr; hsjin@unist.ac.kr; jwyoo@unist.ac.kr

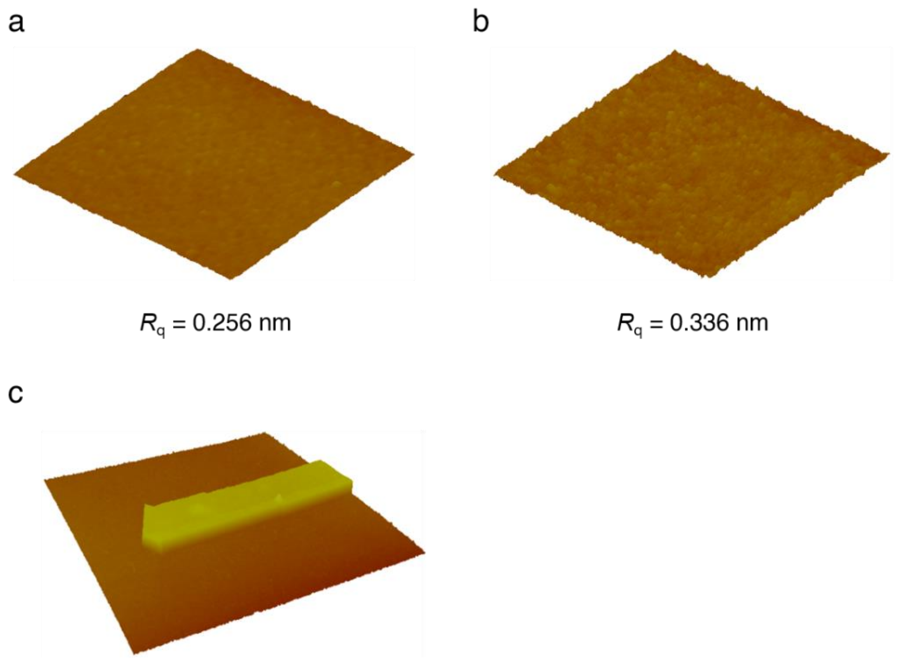

Supplementary Fig. 1 AFM analysis for $PtSe_2$ thin films. a, b AFM images for a pristine $PtSe_2$ film (**a**) and a plasma-treated $PtSe_2$ film (**b**), respectively. The estimated root mean square roughness (R_q) is 0.256 nm for a pristine $PtSe_2$ film and 0.336 nm for a plasma-treated $PtSe_2$ film for the scanned area of 1×1 μm^2. **c** An AFM image for a 10 nm $PtSe_2$ flake. The scanned area is 10×10 μm^2.

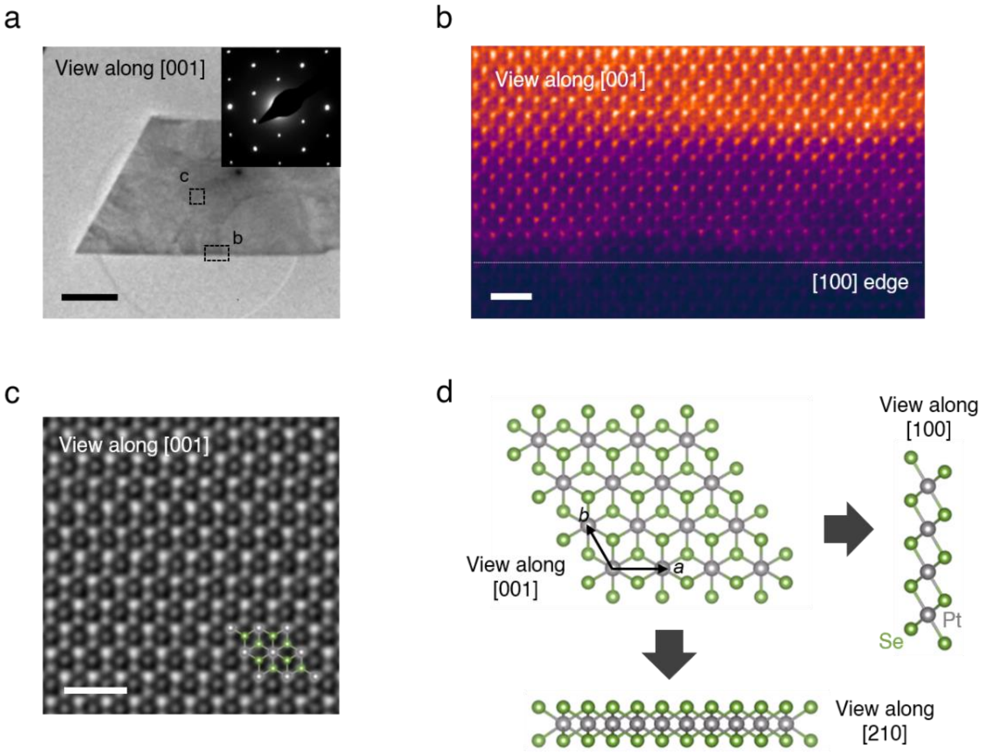

Supplementary Fig. 2 Experimental and atomic modeling of pristine 1T-phase of $PtSe_2$. a A low-magnification TEM image of a pristine $PtSe_2$ flake. An inset is the obtained selected area diffraction pattern, representing the [001] zone axis. **b**, **c** Atomic resolution STEM images of the edge (**b**) and the basal plane (**c**) region of the $PtSe_2$ flake, indicated in (**a**). These images clearly show the hexagonal symmetry of a pristine $PtSe_2$ with inversion symmetry. The elongated edge represents the [100] direction (referred to the zigzag direction). **d** Atomic modeling of a pristine $PtSe_2$ along various zone axes, [001], [210] (=[$11\bar{0}$]), and [100]. Scale bars indicate 500 nm in (**a**) and 1 nm in (**b**, **c**).

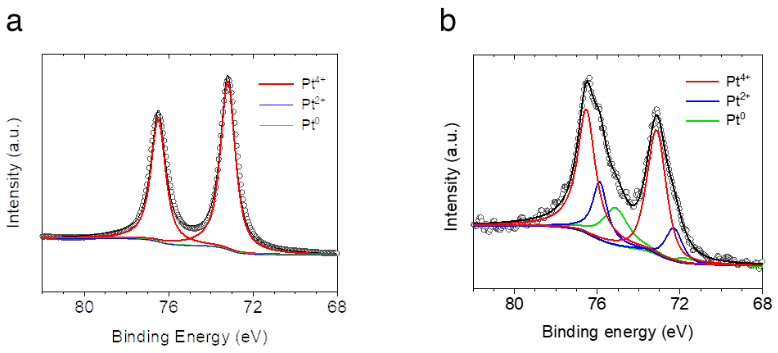

Supplementary Fig. 3 XPS analysis of Pt in $PtSe_2$ thin films with and without plasma treatment. a, b XPS data for a pristine $PtSe_2$ film (**a**) and a plasma-treated $PtSe_2$ film (**b**). The pristine film shows only a Pt^{4+} state, but the plasma-treated film exhibits various Pt states including Pt^{4+}, Pt^{2+}, and Pt^{0}, representing plasma-induced defects. The solid curves are deconvolution fits to the spectra for each state.

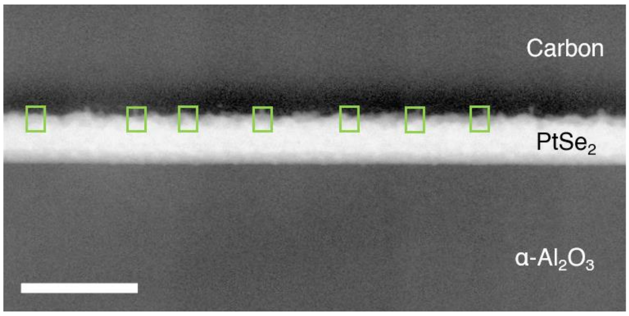

Supplementary Fig. 4 A low magnification TEM image in a plasma-treated $PtSe_2$ film. The green boxes are the 7 different regions used for analyzing a Se/Pt ratio. Their average values for the Se/Pt ratio according to the depth from the surface are shown in Fig. 2b. A scale bar indicates 100 nm.

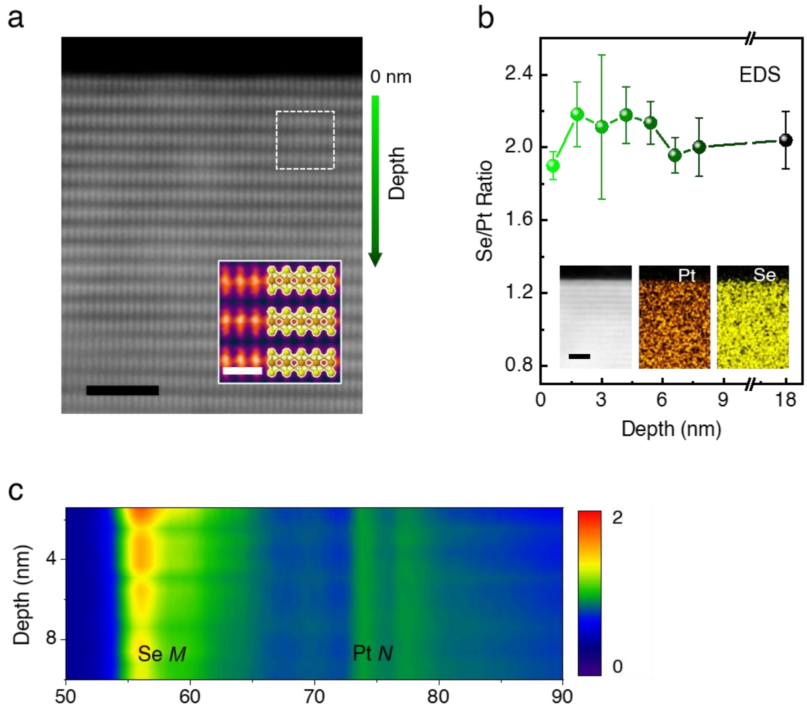

Supplementary Fig. 5 Elemental analysis of a pristine $PtSe_2$ film using STEM. a A cross-sectional HAADF-STEM image of pristine $PtSe_2$ layers. All the layers represent a homogenous and crystalline structure of $PtSe_2$. The inset represents a magnified image of the white dotted box in (**a**) superimposed on a $PtSe_2$ atomic model. Scale bars for (**a**) and the inset are 2 nm and 0.5 nm, respectively. **b** A depth profile of a Se/Pt ratio obtained by EDS measurement. The determined Se/Pt ratio is almost constant as 2, and the error bars result from the deviation of the Se/Pt ratio from 7 different regions. Insets are a HAADF-STEM image and its EDS mapping images showing homogenous distribution of Pt and Se. A scale bars is 2 nm. **c** EELS spectra of Se $M_{4,5}$ and Pt $N_{6,7}$ according to the layer depth. EELS data is normalized to the Pt $N_{6,7}$ peak.

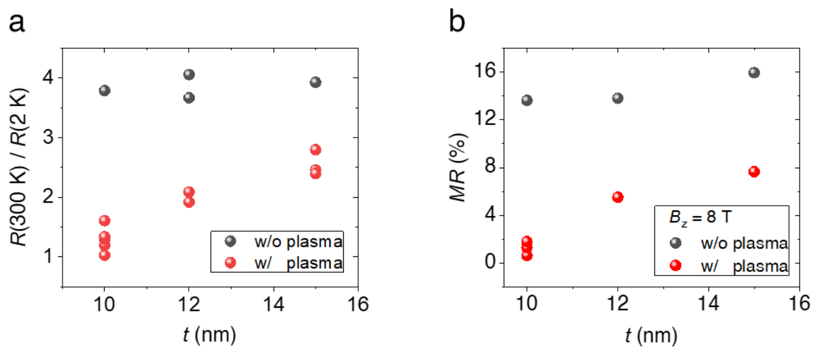

Supplementary Fig. 6 RRR and MR for various thicknesses of $PtSe_2$ films with and without plasma treatment. **a** RRR between 300 K and 2 K and **b** MR under a perpendicular magnetic field measured for the various film thicknesses of $PtSe_2$. Plasma-treated $PtSe_2$ films exhibit significantly reduced RRR and MR compared to those of pristine films. As the thickness of plasma-treated $PtSe_2$ increases, the effect of plasma treatment decreases due to the finite depth of plasma effect.

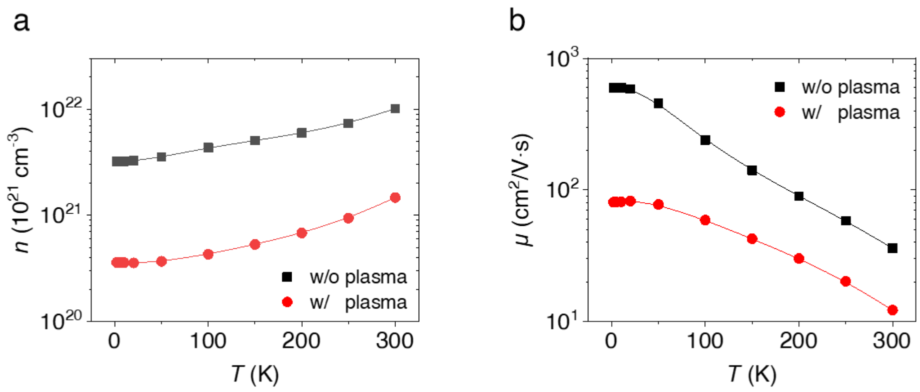

Supplementary Fig. 7 Temperature-dependent carrier concentration and mobility of 10 nm $PtSe_2$ films with and without plasma treatment. a Carrier concentration and **b** carrier mobility from 2 K to 300 K depending on plasma treatment. The estimated Fermi level at 2 K is 0.791 eV at 2 K in a pristine $PtSe_2$ film of 10 nm, while it reduces to 0.185 eV for a plasma-treated $PtSe_2$ film of 10 nm.

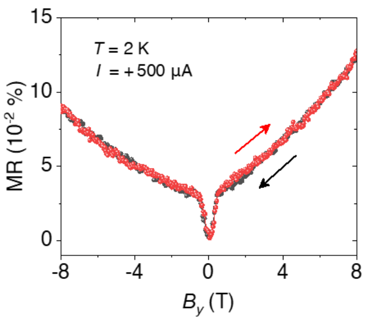

Supplementary Fig. 8 Nonreciprocal MR with reversing a magnetic field sweep in a plasma-treated $PtSe_2$ film of 10 nm. MR results with the opposite field-sweep direction as positive to negative (black) and negative to positive (red). There is no difference between a positive and a negative field sweep direction.

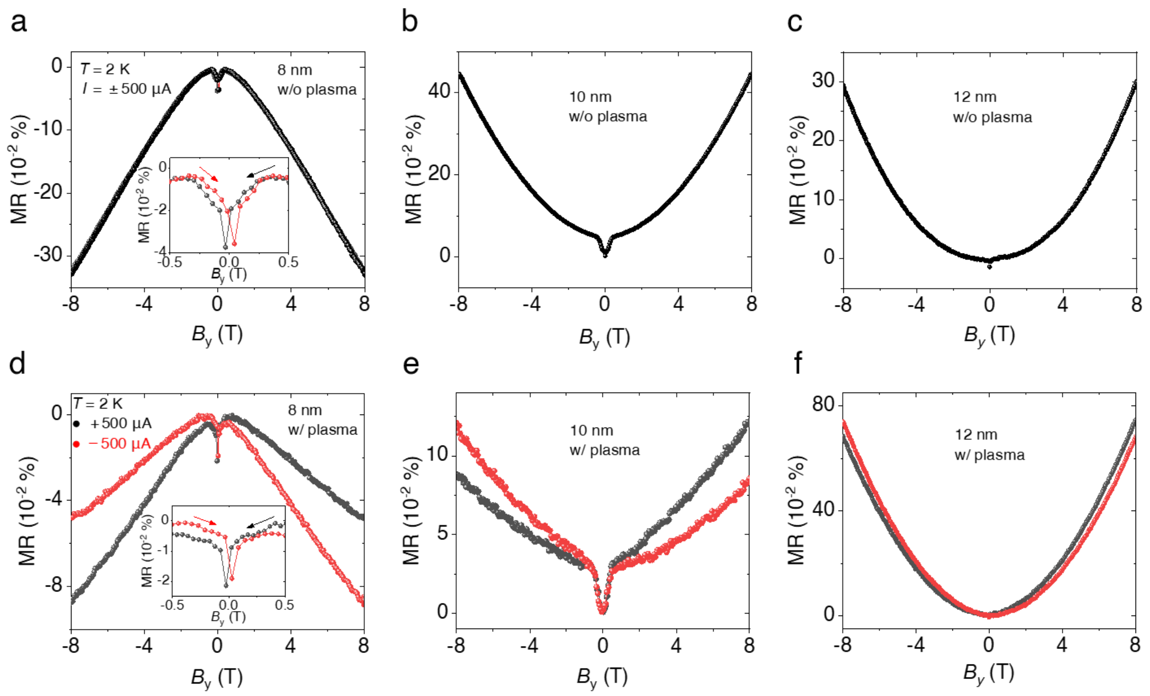

Supplementary Fig. 9 AMR signals near *B* = 0 T in various $PtSe_2$ films at *T* = 2 K. a-c AMR measured for pristine $PtSe_2$ films of 8 nm, 10 nm, and 12 nm. The inset indicates the hysteresis behavior of AMR upon the forward and backward magnetic field sweep. **d-f** AMR measured for plasma-treated $PtSe_2$ films of 8 nm, 10 nm, and 12 nm. For both pristine and plasma-treated samples, the magnitude of AMR decreases with increasing thickness of a $PtSe_2$ film and finally disappears when the thickness get beyond 12 nm.

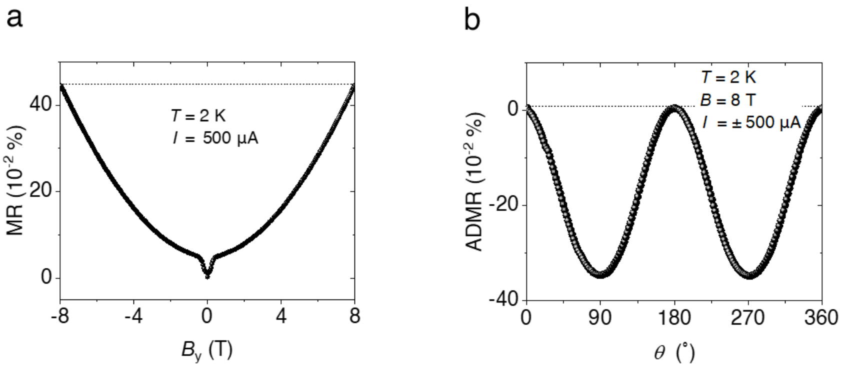

Supplementary Fig. 10 MR for a pristine $PtSe_2$ film of 10 nm at *T* = 2 K. a MR in response to a magnetic field-sweep in the *y*-direction. **b** ADMR measured under the rotation of the applied magnetic field in the *yx* plane. There is no discernable asymmetry in the MR results for a positive and a negative current flow in a pristine $PtSe_2$ film.

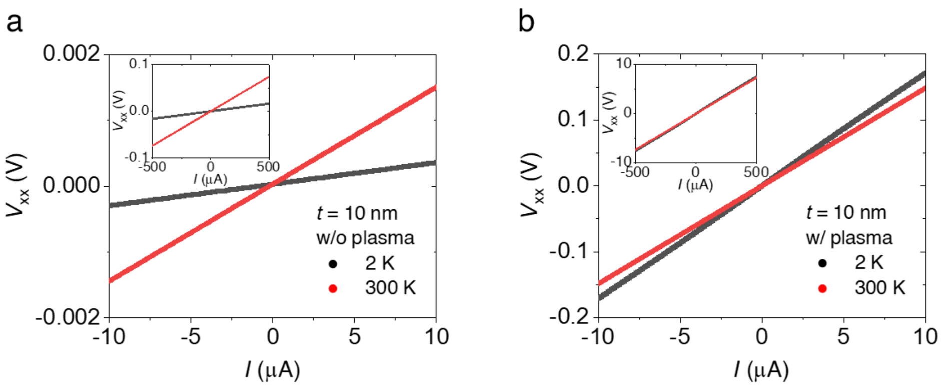

Supplementary Fig. 11 *I-V* characteristics of the 10 nm $PtSe_2$ films with and without plasma treatment. Narrow range *I-V* curves for **a** pristine and **b** plasma-treated $PtSe_2$ films at 2 K and 300 K. The insets show wide range *I-V* curves for the $PtSe_2$ films. Excellent linear behaviors represent the Ohmic contact for both samples.

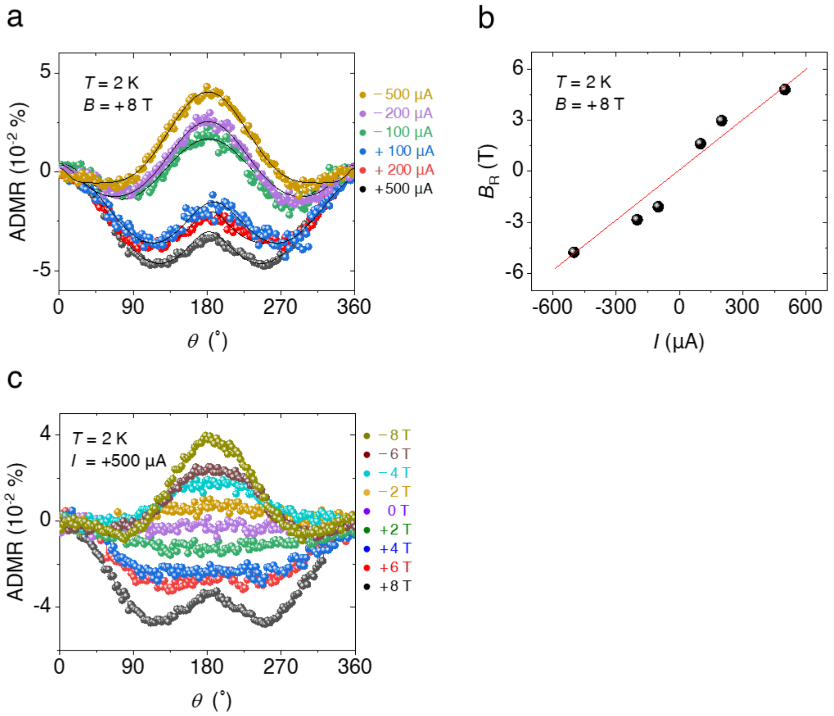

Supplementary Fig. 12 ADMR upon various DC currents and magnetic fields at $T = 2$ K in a plasma-treated $PtSe_2$ film of 10 nm. **a** ADMR in the *yx* plane measured with various applied DC currents. ADMR exhibits asymmetric behaviors between the $+y$ ($\theta = 0^\circ$) and the $-y$ ($\theta = 180^\circ$) direction of a magnetic field. Changing the current direction results in the opposite asymmetric behavior, which becomes stronger with increasing applied currents. **b** The estimated current-induced B_R as a function of applied DC currents. **c** ADMR in the *yx* plane measured with different magnitude of applied magnetic fields.

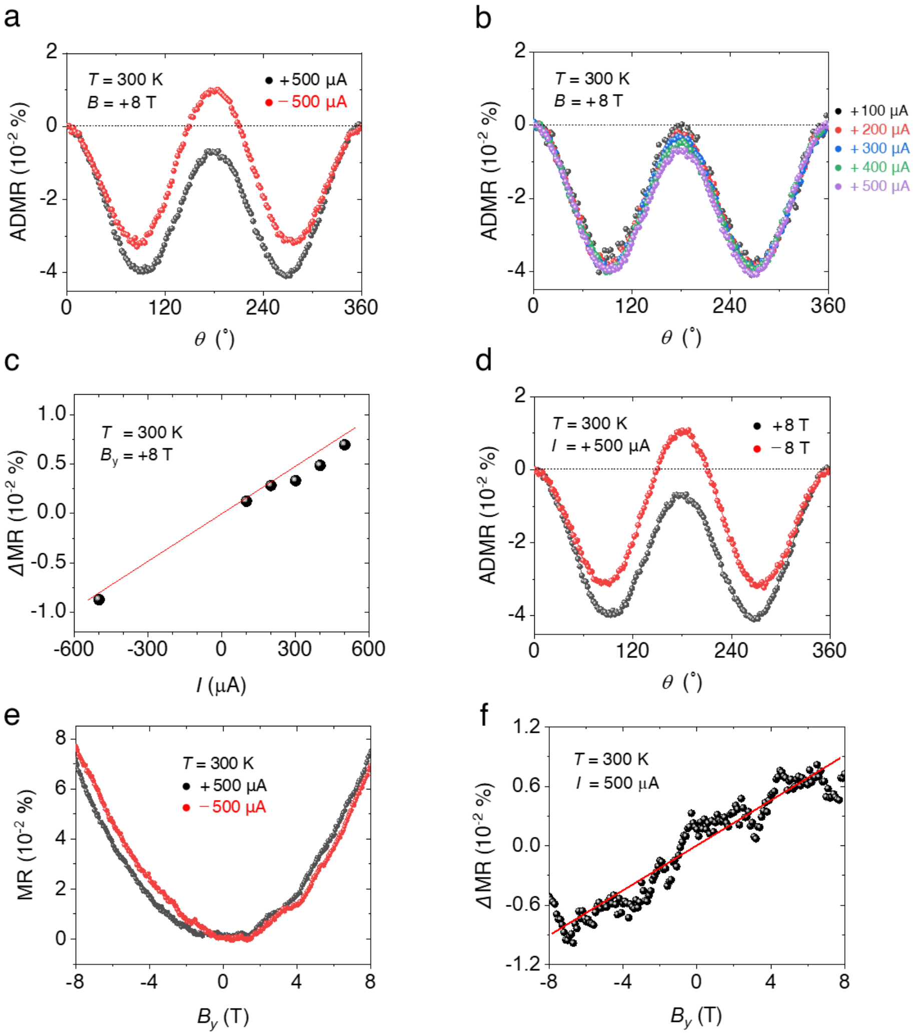

Supplementary Fig. 13 Room-temperature nonreciprocal MR in a plasma-treated $PtSe_2$ film of 10 nm. **a** ADMR in the *yx* plane measured with $B = +8$ T and $I = \pm 500$ μA. **b** ADMR with various currents measured with $B = +8$ T. **c** Nonreciprocal MR depending on a current at $B = +8$ T. Here, ΔMR is collected by the difference MR values at the *y*-directions ($\theta = 0^\circ$ and $\theta = 180^\circ$). **d** ADMR with different magnetic fields with $I = +500$ μA. **e** Nonreciprocal MR measured for $I = \pm 500$ μA. **f** Magnetic field-dependent nonreciprocal MR measured with $I = \pm 500$ μA. Here, ΔMR is determined as MR($+I$) − MR($-I$). All the features follow the nonreciprocal transport relation.

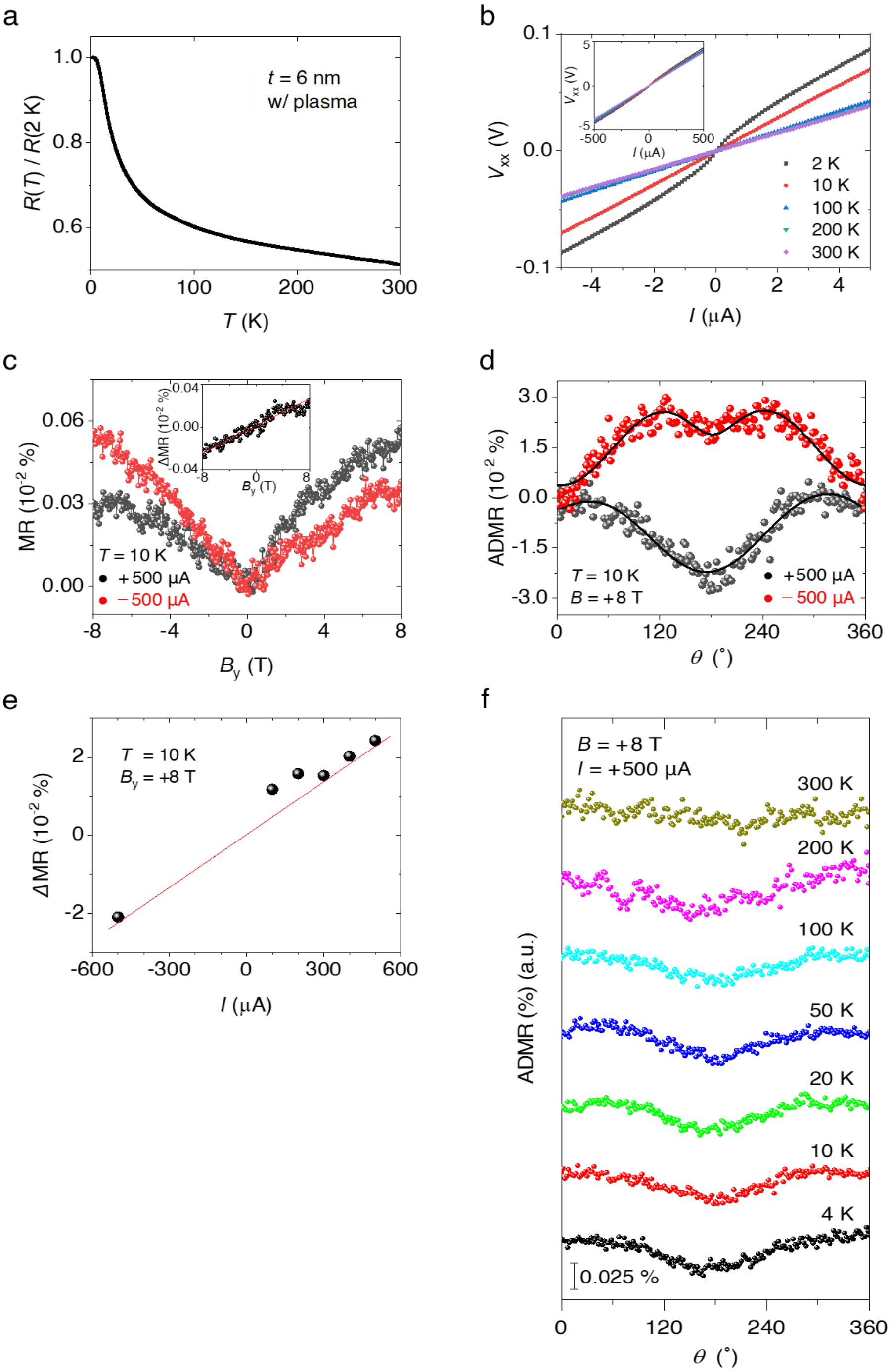

Supplementary Fig. 14 Nonreciprocal MR for a plasma-treated $PtSe_2$ film of 6 nm. a Temperature-dependence resistance. **b** *I*-*V* curves depending on temperature. **c** Nonreciprocal MR measured with $I = \pm 500$ μA at $T = 10$ K. The inset indicates magnetic field-dependent MR collected from the MR values with $I = \pm 500$ μA. **d** ADMR in the *yx* plane measured with $B = +8$ T and $I = \pm 500$ μA at $T = 10$ K. **e** Current-dependent nonreciprocal MR under $B = +8$ T at $T = 10$ K. **f** Temperature-dependent ADMR with $B = +8$ T and $I = +500$ μA.

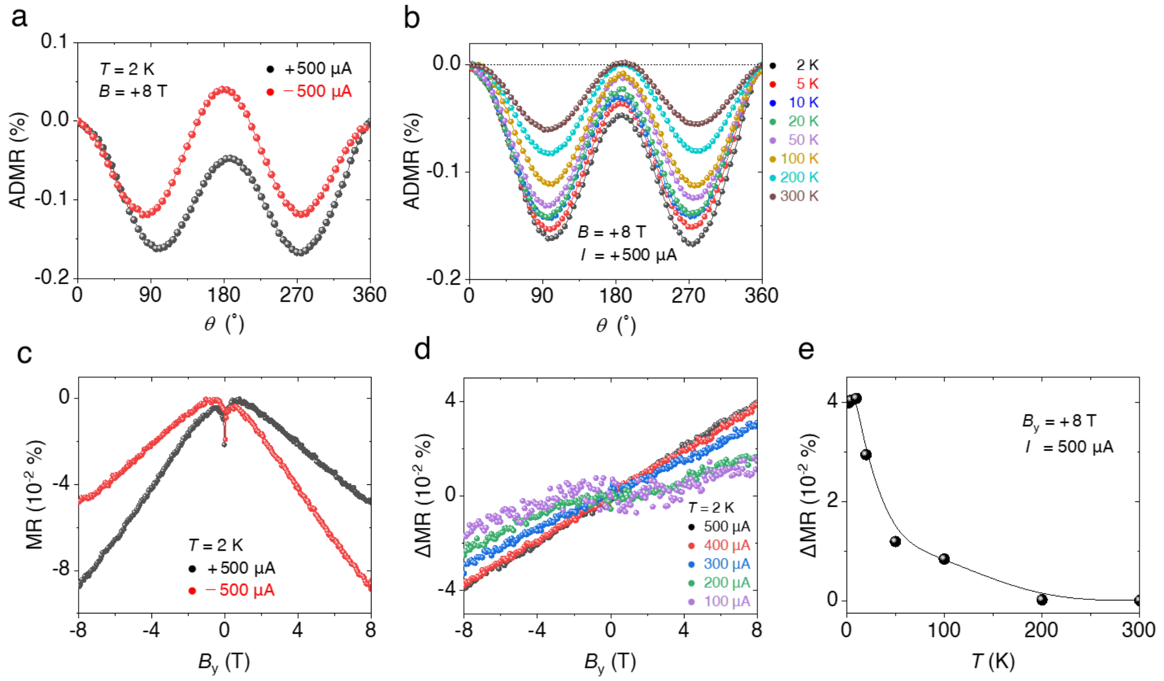

Supplementary Fig. 15 Nonreciprocal MR for a plasma-treated $PtSe_2$ film of 8 nm. a ADMR in the *yx* plane measured with $B = +8$ T and $I = \pm500$ μA at $T = 2$ K. **b** ADMR at various temperatures measured with $B = +8$ T and $I = +500$ μA. **c** Nonreciprocal MR measured with $I = \pm500$ μA at $T = 2$ K. Results exhibit a clear nonreciprocal response. **d** Magnetic field-dependent nonreciprocal MR measured with various DC currents. **e** Temperature-dependent nonreciprocal MR under $B_y = +8$ T.

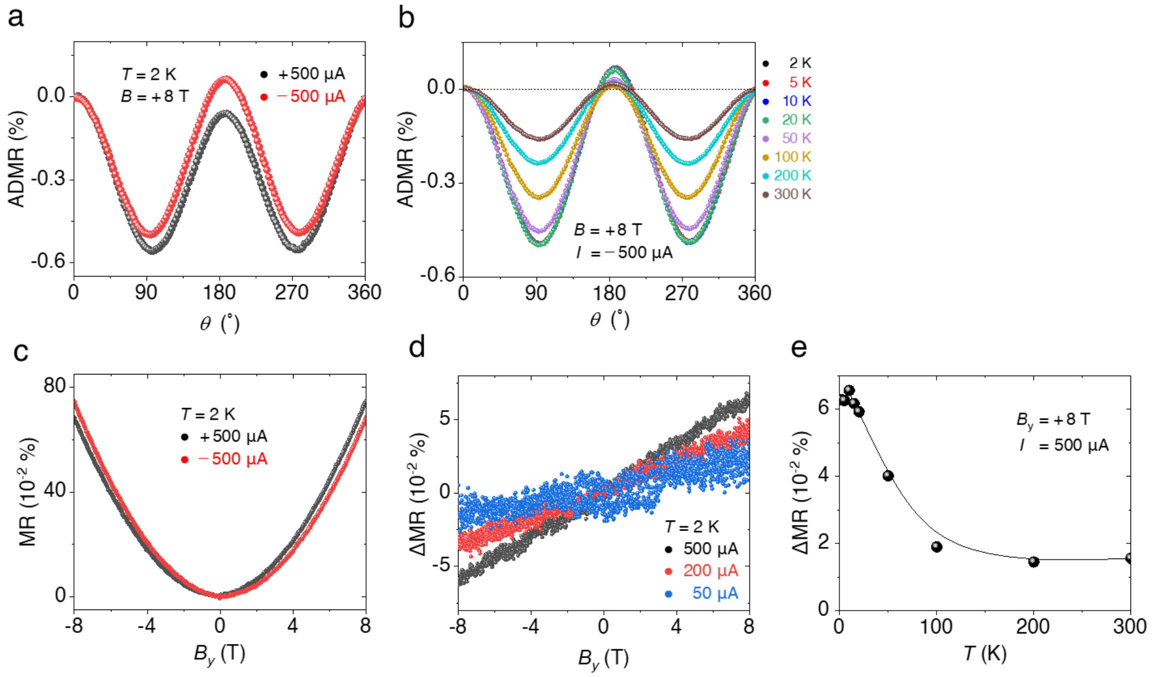

Supplementary Fig. 16 Nonreciprocal MR for a plasma-treated $PtSe_2$ film of 12 nm. a ADMR in the *yx* plane measured with B = +8 T and I = ±500 μA at T = 2 K. There is clear asymmetry at the *y*-directions (θ = 0° and θ = 180°) which shows opposite behavior with reversing a current direction. **b** ADMR at various temperatures measured with B = +8 T and I = −500 μA. As temperature increases, the asymmetry decreases, but it persists up to T = 300 K. **c** Nonreciprocal MR measured with I = ±500 μA at T = 2 K. **d** Magnetic field-dependent nonreciprocal MR measured with various DC currents. **e** Temperature-dependent nonreciprocal MR under B_y = +8 T. The nonreciprocal MR decreases as temperature increases.

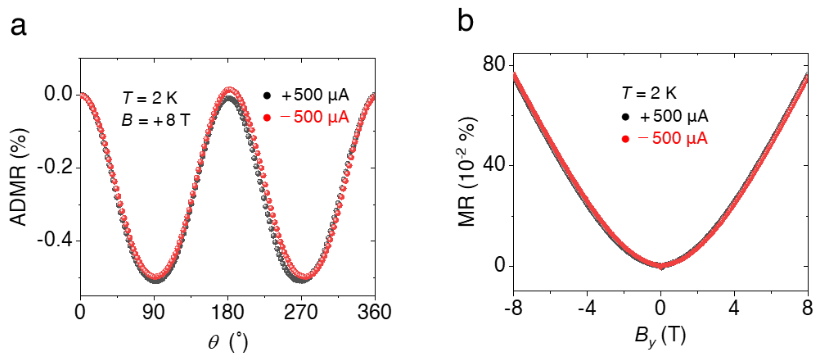

Supplementary Fig. 17 Nonreciprocal MR for a plasma-treated $PtSe_2$ film of 15 nm. a ADMR in the *yx* plane measured with B = +8 T and I = ±500 μA at T = 2 K. Slight asymmetry can be observed in the +*y* (θ = 0˚) and the −*y* (θ = 180˚) direction, and it displays opposite behavior with reversing a current direction. **b** Nonreciprocal MR measured with I = ±500 μA at T = 2 K. MR displays difference between a positive and a negative current, but its magnitude is very small.

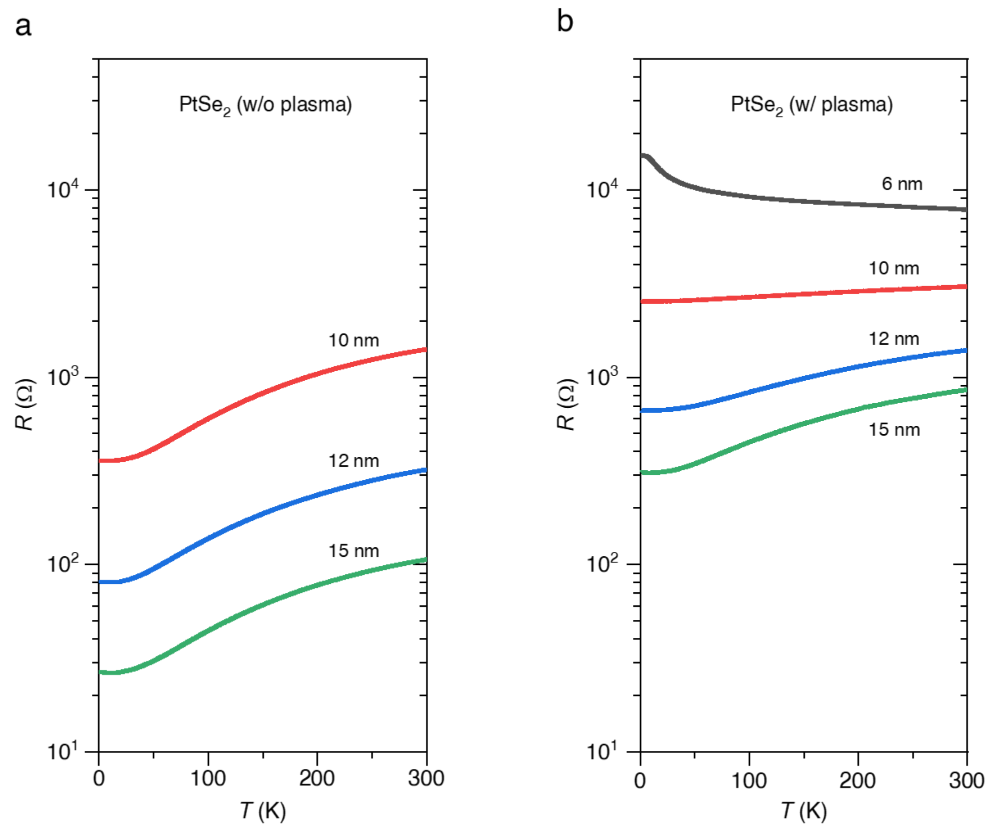

Supplementary Fig. 18 Temperature-dependent resistances of $PtSe_2$ films with and without plasma treatment depending on thickness. Measured longitudinal resistance (R_{xx}) from 2 K to 300 K for **a** pristine $PtSe_2$ films and **b** plasma-treated $PtSe_2$ films. The device structures (i.e. length and width) are same for the measured samples. A pristine $PtSe_2$ flake of 6 nm was not able to be obtained in our experiment.

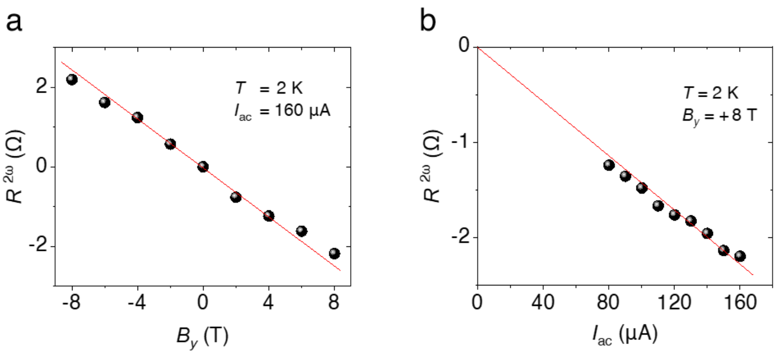

Supplementary Fig. 19 $R^{2\omega}$ with varying a magnetic field and an AC current at T = 2 K for a plasma-treated $PtSe_2$ film of 10 nm. a Magnetic field-dependent $R^{2\omega}$ measured with I_{ac} = 160 μA. Results display linear behaviors. **b** AC current-dependent $R^{2\omega}$ measured under B_y = +8 T. The $R^{2\omega}$ shows linear dependence on the applied AC current.

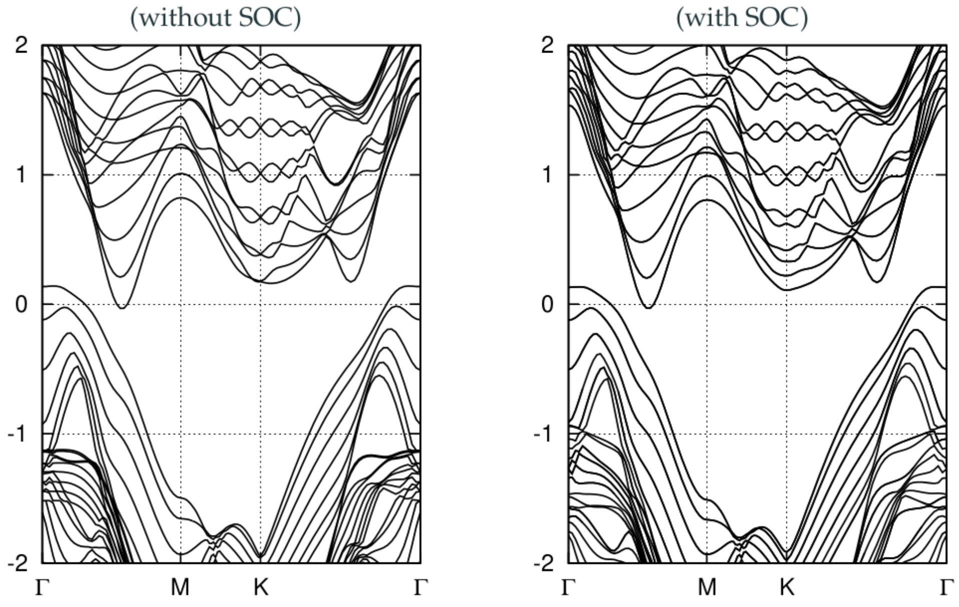

Supplementary Fig. 20 First-principles DFT band structures of the defect-free $PtSe_2$ slab geometry without and with SOC. In the absence of a defect-gradient, the splitting does not appear regardless of the presence or absence of SOC.

$PtSe_2$ without plasma treatment

#	B_R (T)	Nonreciprocal MR_y (10^{-2} %)	RRR (300 K/2 K)	MR_Z (%)	t (nm)
SX-11	-	-	2.40		8
SX-21	-	-	3.79	13.6	10
SX-31	-	-	4.06	13.8	12
SX-32	-	-	3.67		12
SX-41	-	-	3.93	15.9	15

$PtSe_2$ with plasma treatment

#	B_R (T)	Nonreciprocal MR_y (10^{-2} %)	RRR (300 K/2 K)	MR_Z (%)	t (nm)	
					Before plasma treatment	After plasma treatment
SO-01	-	0.02	0.51		22	6
SO-11	1.01	3.9	3.15		25	8
SO-21	4.81	3.2	1.20	0.6	27	10
SO-22	2.98	3.6	1.30	1.3	19	10
SO-31	0.11	0.7	2.12	5.5	45	12
SO-32	0.18	0.6	1.92		35	12
SO-41	0.19	1.0	2.46		30	15
SO-42	0.18	1.1	2.80	7.6	37	15
SO-43	0.19	0.7	2.40		30	15

Supplementary Table 1 Summary of magneto-transport results measured in various $PtSe_2$ samples. Data for a fabricated device name (#), the current-induced effective Rashba field (B_R), nonreciprocal MR measured with DC currents, residual-resistance ratio (RRR) between 300 K and 2 K, magnetoresistance with a perpendicular magnetic field (MR_z), and the thickness of a device (t) before and after plasma treatment. All the results in this table were measured at $T = 2$ K.
